# Metagenomes from Arctic Soil Microbial Communities from the Barrow Environmental Observatory, Utqiaġvik, AK, USA

**DOI:** 10.1128/mra.00528-22

**Published:** 2022-07-18

**Authors:** Neslihan Taş, Baptiste Dafflon, Craig Ulrich, Yuxin Wu, Susannah G. Tringe, Janet K. Jansson

**Affiliations:** a Earth and Environmental Sciences Area, Lawrence Berkeley National Laboratory, Berkeley, California, USA; b Biosciences Area, Lawrence Berkeley National Laboratory, Berkeley, California, USA; c Biological Sciences Division, Pacific Northwest National Laboratory, Richland, Washington, USA; University of Southern California

## Abstract

Here, we report 36 active-layer and 17 permafrost metagenomes from Utqiaġvik, AK, USA. Samples were collected from different topographical features and depths to study Arctic tundra microbiomes.

## ANNOUNCEMENT

Increasing global temperatures are affecting Arctic ecosystems more than any other on the planet (1). With accelerated permafrost thaw, vast Arctic soil carbon stocks (2) are expected to become available for microbial decomposition (3) and result in a positive feedback loop. In this study, we investigated the active-layer (AL) soil and permafrost microbiomes from a coastal Arctic tundra location (4).

AL soils and permafrost were collected from the Next Generation Ecosystem Experiments—Arctic (NGEE-Arctic) research site located in the Barrow Environmental Observatory (BEO), Utqiaġvik, AK, USA (4). The BEO landscape is dominated by different polygon types, representing a gradient of permafrost degradation (4, 5). The AL depth varies between 0.35 and 0.45 m (4). In total, 36 AL samples from organic and mineral layers of polygons were collected in two sampling trips in 2012 (Table 1). Presterilized PVC tubes of 3 cm diameter were inserted into soil incrementally (6), sampling the organic and mineral horizons separately. The soil horizons were visually confirmed; the samples were placed into Whirl-Pak (Madison, WI, USA) bags and flash frozen in liquid nitrogen in the field. The samples were transported to the laboratory in a liquid nitrogen dewar and then stored at −80°C. DNA was extracted as previously described (4).

**TABLE 1:** Sample collection and sequencing data^*a*^

Soil type	Soil horizon	Sampling date		Polygon	Depth (cm)	No. of reads	Avg GC%	Coordinates (°N, °W)	SRA accession no.	JGI taxon object identification no.
		Mo	Yr							
AL	Organic	July	2012	HC-Rep1	7.5	6.9E+07	62.9	71.2, 156.4	SRX1987651	3300001405
AL	Organic	July	2012	HC-Rep2	7.5	1.0E+08	63.9	71.2, 156.4	SRX1987654	3300001418
AL	Organic	July	2012	HC-Rep3	7.5	7.0E+07	63.3	71.2, 156.4	SRX1987657	3300001384
AL	Mineral	July	2012	HC-Rep1	37	7.0E+07	61.6	71.2, 156.4	SRX1987652	3300001409
AL	Mineral	July	2012	HC-Rep2	37	7.1E+07	61.6	71.2, 156.4	SRX1987653	3300001406
AL	Mineral	July	2012	HC-Rep3	37	6.8E+07	61.6	71.2, 156.4	SRX1987658	3300001401
AL	Organic	July	2012	FC-Rep1	10	6.8E+07	61.8	71.2, 156.6	SRX1987241	3300001414
AL	Organic	July	2012	FC-Rep2	10	7.3E+07	58.6	71.2, 156.6	SRX1987457	3300001416
AL	Organic	July	2012	FC-Rep3	10	7.1E+07	62.3	71.2, 156.6	SRX1987459	3300001399
AL	Mineral	July	2012	FC-Rep1	39	8.7E+07	62.2	71.2, 156.6	SRX1987242	3300006055
AL	Mineral	July	2012	FC-Rep2	39	7.3E+07	62.0	71.2, 156.6	SRX1987240	3300001396
AL	Mineral	July	2012	FC-Rep3	39	8.0E+07	60.7	71.2, 156.6	SRX1987244	3300001397
AL	Organic	July	2012	LC-Rep1	15	7.3E+07	61.4	71.1, 156.4	SRX1987639	3300001413
AL	Organic	July	2012	LC-Rep2	15	6.8E+07	58.3	71.1, 156.4	SRX1987638	3300001411
AL	Organic	July	2012	LC-Rep3	15	7.7E+07	51.1	71.1, 156.4	SRX1987646	3300001415
AL	Mineral	July	2012	LC-Rep1	45	6.7E+07	55.4	71.1, 156.4	SRX1987460	3300001410
AL	Mineral	July	2012	LC-Rep2	45	7.1E+07	60.0	71.1, 156.4	SRX4917243	3300001404
AL	Mineral	July	2012	LC-Rep3	45	6.9E+07	58.8	71.1, 156.4	SRX1987640	3300001400
AL	Organic	Sept	2012	HC-Rep1	7.5	5.9E+07	61.5	71.1, 156.4	SRX1987694	3300001454
AL	Organic	Sept	2012	HC-Rep2	7.5	7.2E+07	59.6	71.2, 156.4	SRX1987695	3300001403
AL	Organic	Sept	2012	HC-Rep3	7.5	5.6E+07	58.1	71.2, 156.4	SRX1987724	3300001449
AL	Mineral	Sept	2012	HC-Rep1	37	6.8E+07	61.1	71.2, 156.4	SRX1987692	3300001452
AL	Mineral	Sept	2012	HC-Rep2	37	7.3E+07	59.1	71.2, 156.4	SRX1987693	3300001408
AL	Mineral	Sept	2012	HC-Rep3	37	7.3E+07	58.6	71.2, 156.4	SRX1987723	3300001398
AL	Organic	Sept	2012	FC-Rep1	10	7.2E+07	61.2	71.2, 156.6	SRX1987660	3300001407
AL	Organic	Sept	2012	FC-Rep2	10	6.0E+07	61.3	71.2, 156.6	SRX1987666	3300001383
AL	Organic	Sept	2012	FC-Rep3	10	8.1E+07	61.6	71.2, 156.6	SRX1987663	3300001417
AL	Mineral	Sept	2012	FC-Rep1	39	7.6E+07	62.7	71.2, 156.6	SRX1987659	3300001394
AL	Mineral	Sept	2012	FC-Rep2	39	6.1E+07	60.9	71.2, 156.6	SRX1987665	3300001385
AL	Mineral	Sept	2012	FC-Rep3	39	8.7E+07	61.6	71.2, 156.6	SRX1987664	3300001402
AL	Organic	Sept	2012	LC-Rep1	15	5.1E+07	56.7	71.1, 156.4	SRX1987671	3300001424
AL	Organic	Sept	2012	LC-Rep2	15	6.0E+07	60.6	71.1, 156.4	SRX1987675	3300001427
AL	Organic	Sept	2012	LC-Rep3	15	6.7E+07	61.2	71.1, 156.4	SRX1987696	3300001429
AL	Mineral	Sept	2012	LC-Rep1	45	5.6E+07	53.1	71.1, 156.4	SRX1987674	3300001453
AL	Mineral	Sept	2012	LC-Rep2	45	5.3E+07	59.5	71.1, 156.4	SRX1987672	3300001423
AL	Mineral	Sept	2012	LC-Rep3	45	6.0E+07	60.3	71.1, 156.4	SRX1987677	3300001428
PERM	Mineral	April	2012	FC	55	3.6E+08	55.1	71.3, 156.6	SRX2901267	3300006950
PERM	Mineral	April	2012	FC	151	1.1E+08	48.7	71.3, 156.6	SRX2901268	3300006636
PERM	Mineral	April	2012	FC	183	9.2E+07	63.1	71.3, 156.6	SRX2901418	3300006635
PERM	Mineral	June	2013	FC	76	1.0E+08	55.4	71.1, 156.3	SRX2910105	3300006619
PERM	Mineral	May	2013	LC	258	1.0E+08	56.9	71.2, 156.4	SRX2901419	3300006640
PERM	Mineral	June	2013	FC	292	3.4E+08	55.2	71.1, 156.3	SRX2910045	3300006949
PERM	Mineral	April	2014	HC	23	1.1E+08	59.4	71.1, 156.6	SRX2900831	3300006638
PERM	Mineral	April	2014	FC	36	1.1E+08	59.4	71.2, 156.4	SRX2899914	3300006795
PERM	Mineral	Sept	2014	HC	44	2.8E+08	51.5	71.2, 156.3	SRX2895659	3300006972
PERM	Mineral	April	2014	HC	238	2.8E+08	54.4	71.1, 156.6	SRX2900872	3300006936
PERM	Mineral	April	2014	FC	243	1.5E+08	59.4	71.2, 156.4	SRX2899913	3300006642
PERM	Mineral	April	2014	HC	255	1.2E+08	57.1	71.1, 156.4	SRX2916185	3300006792
PERM	Mineral	Sept	2014	HC	357	3.0E+08	55.3	71.2, 156.3	SRX2896615	3300009010

a AL, active layer; PERM, permafrost; HC, high center polygon; FC, flat center polygon; LC, low center polygon; rep, replicate.

Permafrost cores were collected with a SIPRE soil corer between 2012 and 2014 (7). The corer was augmented with a hydraulic-driven rotary coring platform (Big Beaver; Little Beaver, Inc.) or manually with a gas-powered motor on a 4-m tripod auger. After retrieval, the cores were packed with dry ice in coolers during transport and stored at −25°C in the laboratory. Each permafrost core was first sliced into subsections 10 cm long with a rotary saw in a −18°C room. Then, these subsections were further cut to remove potential surface contaminants via removing the outermost 2 cm using sterile blades on a sonic cutting tool (4). DNA was extracted via the PowerSoil DNA isolation kit (MOBIO, Carlsbad, CA, USA) using 2 g material (Table 1) and quantified using the Qubit double-stranded DNA (dsDNA) high-sensitivity (HS) assay (Invitrogen, Carlsbad, CA, USA).

Metagenomic sequencing was performed at the DOE Joint Genome Institute (JGI). AL soils were submitted for regular DNA library preparation and sequenced using the Illumina HiSeq 2000 platform (2 × 150 bp; Table 1). Permafrost samples were submitted for low-input DNA library preparation and sequenced using the Illumina HiSeq 2500 platform (2 × 150 bp; Table 1). BBDuk was used to remove known Illumina adapters, low-quality (>Q12) reads, spike-ins or phiX, and reads <51 bp long (8). *De novo* annotation of unassembled and SPAdes-assembled (9) sequences was done in JGI’s Integrated Microbial Genomes & Microbiomes (IMG/M) system (10). These data provide a resource to analyze microbial functions across topographical features and at different depths at a changing Arctic tundra site in order to understand and predict future climate trends and threads (11–13).

### Data availability.

The sequences were deposited at the National Center for Biotechnology Information (NCBI) database under the SRA accession numbers listed in Table 1. The sequences and annotations are available under JGI IMG/M proposal ID 1044. A summary of the scaffolds and genes can be found at https://doi.org/10.6084/m9.figshare.20164358.v1.
